# Expenditures on vaccine-preventable disease surveillance: Analysis and evaluation of comprehensive multi-year plans (cMYPs) for immunization

**DOI:** 10.1016/j.vaccine.2018.07.068

**Published:** 2018-10-29

**Authors:** Azfar Hossain, Claudio Politi, Nikhil Mandalia, Adam L. Cohen

**Affiliations:** Expanded Programme on Immunization (EPI), Department of Immunizations, Vaccines, and Biologicals (IVB), World Health Organization (WHO), Avenue Appia 20, 1211 Genève 27, Switzerland

**Keywords:** Disease surveillance, Cost, Expenditure, Vaccine-preventable disease, Immunisation, cMYP

## Abstract

Despite the importance of vaccine-preventable disease (VPD) surveillance, little is known about the costs of monitoring disease. We used Comprehensive Multi-Year Plans for Immunization (cMYPs) – developed by countries following guidelines from the World Health Organization and United Nations Children’s Fund – to estimate expenditures on VPD surveillance at the country level in 2015 US Dollars (USD) in 63 low- and middle-income countries. To evaluate the reliability of cMYP estimates, we also compared cMYP data with findings from previous research studies and assessed whether countries explicitly budgeted for major categories of surveillance activities in their plans for immunization. According to our analysis of cMYPs, countries spent an annual median of $406,108 on VPD surveillance ($0.04 per capita and $1.47 per infant), with reported expenditures ranging from $1,098 (Kiribati) to $21,644,770 (Nigeria). However, the majority of countries failed to explicitly mention several key categories of surveillance activities in their plans, especially laboratory-related surveillance activities. Our results show a large amount of variation in surveillance expenditures (total, per capita, and per infant) between countries and provide insights to improve costing guidelines and practices.

## Highlights

• Large range of annual VPD surveillance expenditures in 63 lower-income countries.
• Median expenditure on VPD surveillance was $406,108 ($0.04 per capita) in 2015 USD.
• Countries allocated a median of 4.3% of routine immunization funds on surveillance.
• Most countries did not specify if all surveillance needs were included in budgets.
• Findings can inform efforts to improve surveillance costing methodologies.

## 1 Introduction

Infectious disease surveillance is used to monitor levels of disease, identify where interventions are needed, and evaluate the impact of vaccination and other activities [1]. Strong surveillance systems provide valuable benefits, from informing vaccination strategies to protect populations against communicable illness, to helping bring diseases like poliomyelitis to the verge of eradication [1]. For vaccine-preventable diseases (VPDs), surveillance is needed to describe disease burden to inform decisions around vaccine introduction; to measure the impact of vaccines used in the Expanded Programme on Immunization (EPI); to document the control, elimination, and eradication of pathogens; and to identify outbreaks that may trigger reactive vaccination campaigns. To understand the costs of VPD surveillance is of particular concern in low- and middle-income countries, where there may be limited resources for public health activities such as surveillance and laboratory capacity.

Despite consensus on the importance of monitoring communicable illnesses, little is known about how much countries should spend on surveillance activities, and very few studies have attempted to quantify surveillance expenditures for VPDs. Wolfson et al. and Gandhi et al. projected how much additional spending was needed on VPD surveillance globally as part of analyses of the Global Immunization Vision and Strategy and Global Vaccine Action Plan, but these studies did not assess current national VPD surveillance expenditures [2], [3]. Irurzun-Lopez et al. calculated and compared 2012 costs of meningitis surveillance in Niger and Chad; they found that Niger spent four times more per capita ($0.12 versus $0.03) and enjoyed a more reliable detection system [4]. Lukwago et al. and Somda et al. determined annual surveillance costs of Integrated Disease Surveillance and Response (IDSR) systems in Uganda, Burkina Faso, Eritrea, and Mali and similarly found a large range in expenditures (less than $0.01–$0.09) between nations, while Toscano et al. calculated the annual cost of implementing VPD surveillance at a sentinel hospital in Costa Rica ($420,000) [5], [6], [7]. However, these studies encompass very few countries, and surveillance requirements in one setting may not be generalizable to other contexts. Improving our current understanding of surveillance costs is important, as countries need to mobilize resources to effectively conduct surveillance.

In this paper, we analyzed levels of spending on VPD surveillance by turning to a previously unexplored source: Comprehensive Multi-Year Plans for Immunization (cMYPs), prepared by low- and middle-income countries according to World Health Organization (WHO) and United Nations Children’s Fund (UNICEF) guidelines [8]. Through our study, we aimed to provide a picture of the current global surveillance expenditure landscape and to evaluate current budgeting practices used to plan for surveillance activities in cMYPs. An understanding of how much countries currently spend on surveillance – and how reliable those estimates may be – is an essential first step to determining the true costs of fully functional VPD surveillance systems in settings around the world.

## 2 Methods

### 2.1 cMYP overview

Ministries of health develop cMYPs to plan for and budget all immunization-related activities, including VPD surveillance. Each nation’s cMYP has two main components: a programmatic document and the Costing and Financing Tool. The programmatic document describes a country’s current VPD burden, existing immunization resources (including surveillance systems), and objectives to improve child health indicators. In the Excel-based Costing and Financing Tool, the country reports all immunization-related expenditures (in US Dollars or local currency) from one previous baseline year and projects expected costs for the next five years [8]. The Tool includes a specific table outlining surveillance expenses for VPDs such as polio, measles and tetanus (although countries rarely indicate which specific VPDs are accounted for by these expenses), and additional surveillance-related costs can often be found in separate sections for immunization personnel, transport, equipment, and overhead. Countries also provide important demographic and financial information in this part of the cMYP, including their total population, annual number of births, and annual spending on both routine and supplemental immunization activities [8].

The following analysis made use of expenditure data from baseline years reported in cMYP Costing and Financing Tools, as these reflect actual expenditures incurred by countries.

### 2.2 Sample and sampling method

Supplementary data associated with this article can be found, in the online version, at https://doi.org/10.1016/j.vaccine.2018.07.068.

Appendix 1 provides a list of all sampled countries and their baseline years. Our analysis included 63 out of all 64 countries that published cMYPs providing baseline costs (reported expenditures from the year before a cMYP is developed) as of August 2016. Democratic People’s Republic of Korea was excluded from the study because neither WHO nor the World Bank provided the country’s GDP deflator values needed for our analysis. While baseline years ranged from 2004 to 2015, 56 (89%) of the 63 countries had baseline years of 2009 or after.

**Supplementary data 1:** 

cMYPs were accessed through the WHO website [9]. In cases where a country had multiple cMYPs, only the most recent cMYP was assessed.

### 2.3 Calculating country-level surveillance expenditures and metrics

For every country, we first recorded the total baseline year spending on VPD surveillance activities listed in the Costing and Financing Tool’s surveillance expenditure table (Table 8.4 in the Costing and Financing Tool version 3.8.3). This table budgets for activities and resources that fall under one of the following surveillance areas: Detection and Notification, Case and Outbreak Verification and Investigation, Data Management, Laboratory, and Supportive Activities. We then screened the rest of the Tool to identify any additional surveillance expenditures listed in other sections, such as salaries of VPD surveillance officers listed in the “Personnel Costs” section. All relevant expenditures were added together to calculate the country’s total expenditure on VPD surveillance during its baseline year.

After extracting the country’s total spending on VPD surveillance, we used additional information provided by the cMYP to calculate the following indicators to facilitate cross-country comparisons. We calculated the surveillance expenditure per capita by dividing the total surveillance expenditure by the country’s total population, as well as the surveillance expenditure per infant by dividing the total surveillance expenditure by the country’s annual number of births. We also determined the percentage of routine immunization expenditures allocated to surveillance by dividing the country’s spending on surveillance by its spending on routine immunization activities. When considering routine immunization expenditures, we did not consider spending on vaccination campaigns or shared health system costs, to facilitate cross-country comparability and to remain consistent with global costing methodologies (for instance, the definition of the indicator “total expenditure on routine immunization” according to the WHO-UNICEF Joint Reporting Form) [10].

Because baseline years ranged from 2004 to 2015, we converted all surveillance expenditures (total, per capita, and per infant) to 2015 US Dollar (USD) values using 2015 GDP deflators. 2015 GDP deflators for 61 out of 63 countries were obtained from the World Bank website [11]. Since the World Bank did not provide recent deflators for Eritrea and Somalia, we converted these countries’ surveillance expenditures to 2014 USD values using 2014 GDP deflators from WHO [12].

### 2.4 Evaluating reliability of cMYP estimates

To evaluate the reliability of cMYP expenditure estimates, we compared cMYP data with the findings of the study conducted on the 2012 costs of meningitis surveillance in Chad and Niger (Irurzun-Lopez et al.). For the comparisons, we used 2012 projected data for Niger and 2013 baseline expenditures for Chad. All expenditures (from the cMYPs and the previous study) were converted to 2015 USD values for consistency with the rest of our analysis.

While screening each country’s cMYP, we also recorded if the Costing and Financing Tool explicitly reported expenditures for each of the major categories of surveillance activities: personnel; transport; laboratory personnel; laboratory reagents; other laboratory equipment and supplies; and laboratory overhead. These are the main cost categories listed in previous studies and in SurvCost, a tool developed by the Centers for Disease Control and Prevention (CDC) to calculate surveillance costs within countries [4], [6], [7], [13].

## 3 Results

### 3.1 Country-level surveillance expenditures and metrics reported in cMYPs

**Fig. 1:**
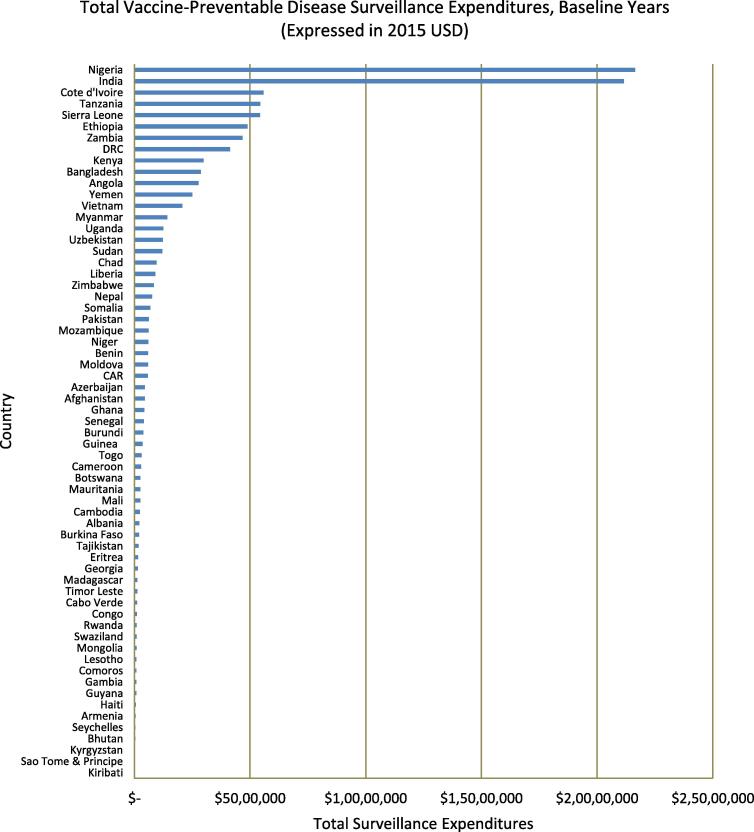
Total annual expenditures on VPD surveillance per country during baseline years, expressed in 2015 USD (except those for Eritrea and Somalia, which are in 2014 USD). Expenditures ranged from $1098 in Kiribati to $21,644,770 in Nigeria.

**Fig. 2:**
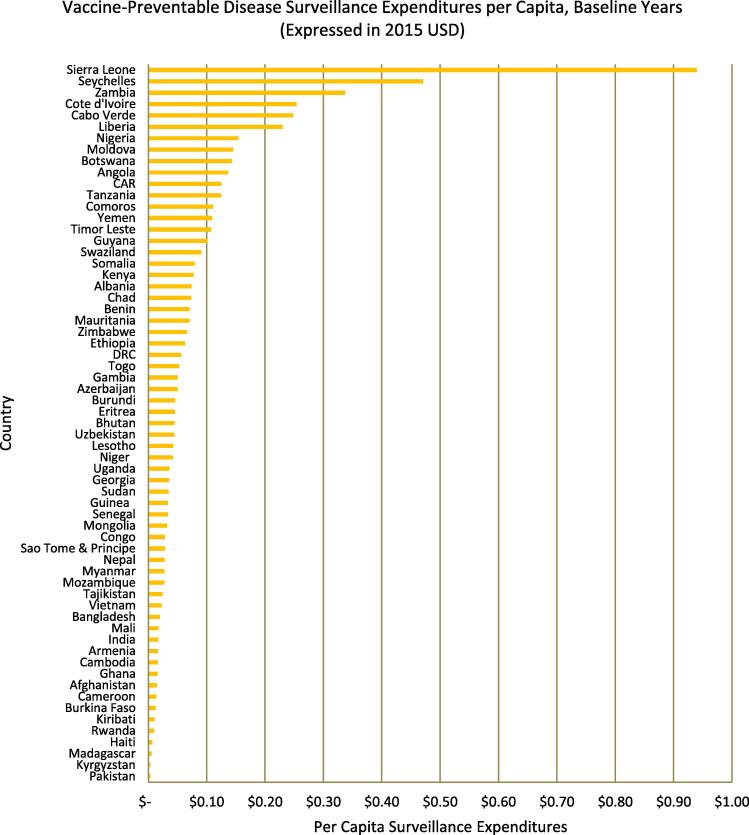
Per capita VPD surveillance expenditures during baseline years, expressed in 2015 USD (except those for Eritrea and Somalia, which are in 2014 USD). Expenditures ranged from less than $0.01 per capita in Pakistan to $0.94 per capita in Sierra Leone.

**Fig. 3:**
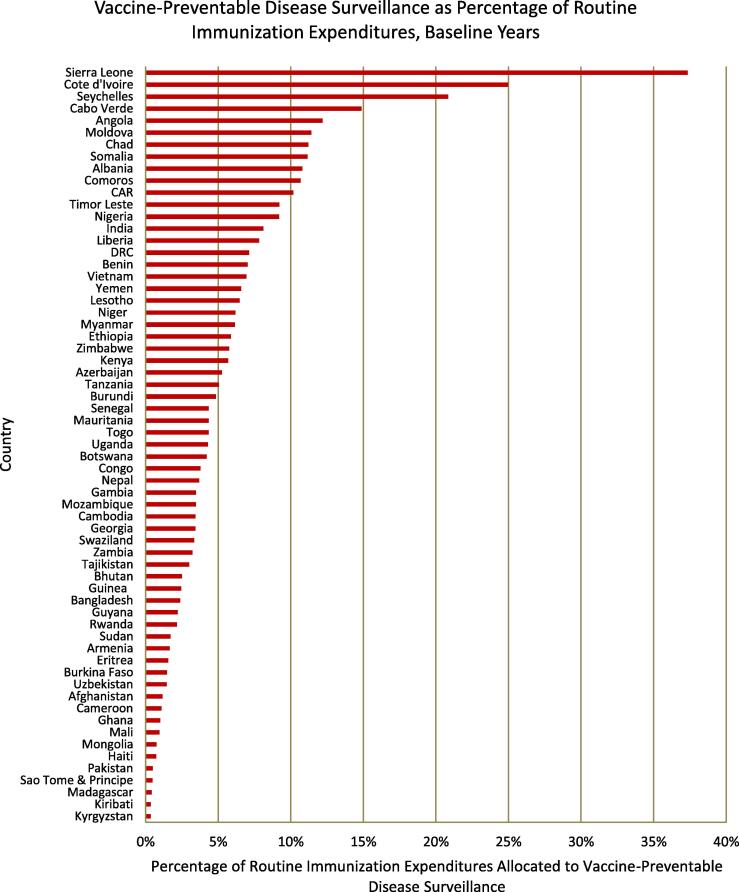
Percentage of routine immunization expenditures allocated to VPD surveillance during baseline years. Routine immunization expenditures do not include spending on vaccination campaigns or shared health system costs.

After all baseline year expenditures were converted to 2015 USD values, we found that countries spent a median^1^ of $406,108 per year on VPD surveillance, with annual expenditures ranging from $1098 (Kiribati) to $21,644,770 (Nigeria) (Fig. 1). Nigeria and India recorded higher surveillance expenditures than all other countries, with both spending over three times more than Cote d’Ivoire, the country which recorded the third greatest expenditures. The median spending per capita and per infant were $0.04 (Georgia, Uganda, Niger, Lesotho, Uzbekistan, and Bhutan) and $1.47 (Lesotho), respectively, once again with a large variation between countries (Fig. 2). While three countries – Seychelles, Cote d’Ivoire, and Sierra Leone – reported using more than 20% of routine immunization funds on VPD surveillance activities, over half (36 of 63) of the countries examined reported that surveillance accounted for less than 5% of routine spending (Fig. 3).

Table 1 shows mean and median VPD expenditures per capita among countries of different population sizes. While countries with populations of less than 1 million spent a mean of $0.14 per capita on VPD surveillance, mean expenditures dropped to $0.10 per capita for countries with populations between 1 and 10 million, $0.07 per capita for countries with populations between 10 and 30 million, and $0.05 per capita for those with over 30 million people.

**Table 1:** Vaccine-preventable disease (VPD) surveillance expenditures per capita by population.

Population size, baseline year	Number of countries in sample	Mean VPD surveillance expenditures per capita, baseline year (expressed in 2015 USD)	Median VPD surveillance expenditures per capita, baseline year (expressed in 2015 USD)
Less than 1 million	7	$0.14	$0.10
1–10 million	24	$0.10	$0.05
10–30 million	19	$0.07	$0.03
More than 30 million	13	$0.05	$0.03
All countries	63	$0.09	$0.04

See Appendix 1 for data from all countries.

### 3.2 Evaluating reliability of cMYP estimates

Table 2 provides 2012 surveillance expenditures for Niger and Chad expressed in 2015 USD values. We observed a discrepancy in expenditures for Niger between the country’s cMYP and the study by Irurzun-Lopez et al. which used the ingredients approach (listing all needed resources, determining the quantity and unit cost of each, and summing expenses to determine the total cost of surveillance) [8]. Spending on all VPD surveillance according to the cMYP ($636,444, or $0.04 per capita), which would include multiple diseases, was less than one third of the cost of only meningitis surveillance calculated by Irurzun-Lopez et al. ($1,992,487, or $0.12 per capita) [4]. We found more alignment between the two sources in expenditures for Chad: the cMYP indicated $951,589 ($0.07 per capita) was spent on all VPD surveillance, while the case study reported $299,754 ($0.03) was spent on meningitis surveillance [4].

**Table 2:** Niger and Chad surveillance expenditures according to Comprehensive Multi-Year Plans for Immunization (cMYPs) versus Irurzun-Lopez et al. (US$).^A^

	Niger		Chad	
	cMYP (all VPDs)^a^	Irurzun-Lopez et al. (Meningitis)^b^	cMYP (all VPDs)^c^	Irurzun-Lopez et al. (Meningitis)^b^
Total expenditure on surveillance	$636,444	$1,992,487	$951,589	$299,754
Per capita expenditure on surveillance	$0.04	$0.12	$0.07	$0.03

A This table evaluates the reliability of cMYP estimates by comparing them to expenditure findings by Irurzun-Lopez et al.

a 2012 cMYP *projected* expenditures (not reported baseline year expenditures) for all vaccine-preventable disease surveillance in Niger, converted to 2015 USD.

b 2012 costs of meningitis surveillance calculated using ingredients method, converted to 2015 USD.

c 2013 baseline year *reported* expenditures for all vaccine-preventable disease surveillance in Chad, converted to 2015 USD.

Table 3 shows the percentages of countries that explicitly mentioned major cost categories of surveillance in their Costing and Financing Tools (country-specific information is available in Appendix 2). While 48 out of 63 countries (76%) explicitly budgeted for surveillance personnel, only 23 (37%) provided expenditures on laboratory personnel, and only 9 (14%) and 14 (22%) did so for laboratory reagents and overhead, respectively. No country explicitly mentioned all six major surveillance cost categories in its cMYP.

**Supplementary data 2:**

**Table 3:** Major cost categories of surveillance in Comprehensive Multi-Year Plans for Immunization (cMYPs).^a^

Surveillance cost category	Number of cMYPs (n = 63) that explicitly budgeted for cost category
Surveillance-specific personnel	48 (76%)
Surveillance-specific transport	32 (51%)
Laboratory personnel	23 (37%)
Laboratory reagents	9 (14%)
Other laboratory equipment and supplies	36 (57%)
Laboratory overhead	14 (22%)

a Data in this table come from countries’ cMYPs.

## 4 Discussion

Like previous studies by Irurzun-Lopez et al. and Somda et al. which used different costing methodologies, we observed large differences in VPD surveillance expenditures (total, per capita, and per infant) reported in cMYPs between countries [4], [6]. This finding matches expectations, as there is a large amount of variation in the types of surveillance conducted and how surveillance is costed, and more developed surveillance systems are expected to incur larger costs. For example, high annual spending on surveillance in Nigeria ($21,644,770, or $0.15 per capita, in 2015 USD) and India ($21,158,601, or $0.02 per capita, in 2015 USD) likely results from their large populations and need for intense polio surveillance, as both countries have until recently had ongoing transmission of poliovirus. The Global Polio Eradication Initiative (GPEI) reports that polio surveillance costs $17 million annually in Nigeria and $7.20 million annually in India, which likely accounts for many of the disease monitoring expenses listed in their cMYPs [14].

As shown in Table 1, we observed a negative relationship between population size and per capita VPD surveillance expenditures: average spending per capita tended to decrease as countries became larger. This may demonstrate efficiency gains that countries with larger populations (and perhaps higher population densities) are able to achieve through exploiting economies of scale.

Although we also noted outliers when analyzing spending per capita and per infant, these countries’ cMYPs did not fully explain their surveillance expenditures nor how the expenditures were estimated. For example, Zambia reported spending $0.34 per capita (in 2015 USD) on VPD surveillance during its baseline year (2012), over eight times the median value. While this cost may relate to surveillance preparations ahead of new vaccine introductions (Zambia introduced the pneumococcal conjugate vaccine and Rotavirus vaccine the following year, 2013), its cMYP did not mention any such preparations, nor specify what other resources accounted for the relatively high surveillance cost [15]. In contrast, Pakistan reported spending less than $0.01 per capita during its baseline year (also 2012), an unexpected finding given its status as a polio-endemic country. Although Pakistan’s relatively low per capita expenditures may simply suggest that it did not make significant new investments in VPD surveillance during the reporting year (perhaps because major investments were actualized either before or after 2012), it could also indicate a failure to record all surveillance expenditures in the country’s cMYP. More research on the surveillance activities of both high- and low-spending countries is needed to further understand the large spectrum of expenditures found in our assessment.

Despite this large variation in expenditures, surveillance received a relatively small portion of routine immunization funds (a median of 4.3%) in almost all settings. This could signal that country stakeholders may not prioritize budgeting for surveillance in comparison to other vaccination-related activities, despite its relative importance – or that surveillance may cost less than other immunization activities. It may also reflect the methodological difficulty of separating surveillance costs from immunization and health system costs, as many health resources are shared across several programs and only a few are exclusive to VPD surveillance.

When comparing cMYP data to the case study by Irurzun-Lopez et al., the cMYPs should report higher expenditures because they considered spending on surveillance for all VPDs, while the case study only considered meningitis surveillance. Consequently, Niger’s cMYP does not appear to reflect surveillance expenditures consistent with the case study. The cMYP projected Niger would spend $0.04 per capita on all VPD surveillance in 2012, but Irurzun-Lopez et al. reported the country spent $0.12 per capita on meningitis surveillance alone that year [4]. In contrast, Chad’s cMYP data seem more aligned: the reported expenditure on all VPD surveillance ($0.07 per capita) was greater than the cost of meningitis surveillance ($0.03 per capita) calculated in the case study [4].

Although Irurzun-Lopez et al. reported that they may have slightly overestimated costs in Niger, minor miscalculations probably cannot account for the large discrepancy between the cMYP and case study values [4]. Assuming that the estimates provided by Irurzun-Lopez et al. reflect true expenditures, our observations suggest that certain nations (like Chad) may include relatively accurate costs of monitoring VPDs within their cMYPs, but others (like Niger) may significantly underestimate actual expenditures, potentially increasing their risk of underfunding surveillance. Such disparities between cMYPs likely result from countries employing different costing methodologies when developing these plans. Some countries use an ingredients approach in cMYPs to calculate surveillance expenditures, as did Irurzun-Lopez et al. However, other countries’ cMYPs may simply assign a lump sum of funds to surveillance activities based on historical budget allocations (which may no longer be accurate as surveillance needs and capacities change over time); again, since surveillance comprises a relatively small portion of routine immunization expenditures, it may not receive appropriate budgeting priority (A. Colombini, personal communication, August 2016). While neither Chad nor Niger specified their method used to calculate cMYP expenditures, we noted that Niger’s Tool did not clearly report spending on laboratory personnel, reagents, and overhead, and this could explain the apparent underestimation of expenditures. Additional country case studies are needed to further assess the accuracy of surveillance expenditures that countries list in their cMYPs, as well as to better understand the costs associated with different types of surveillance systems and implementation modalities.

More generally, most countries failed to explicitly mention several major categories of surveillance expenditures, especially laboratory-related surveillance requirements. Less than half of countries listed laboratory staff salaries in their budgets and less than one third budgeted for reagents, two key needs identified by previous studies as significant expenses for VPD surveillance [4], [6], [7], [13]. While surveillance-specific personnel and transport appeared more regularly in cMYPs, none of these requirements were referenced by more than 76% of countries. When a cMYP does not explicitly mention one of these resources, we cannot determine if the expenditure was budgeted under a broader category or if it was overlooked completely. Therefore, while some cMYPs may have included all major expenses, many countries likely fail to include the full costs of VPD surveillance.

Several factors contribute to this lack of clarity and should be addressed. Many cMYPs use broad labels for surveillance activities (such as “Support for laboratory operations”), making it impossible to determine which costs are actually included. Countries should use clearer descriptions of requirements – like CDC’s SurvCost tool – to indicate which needs have actually contributed to spending [13]. In addition, laboratory costs are often recorded using a different planning tool than the cMYP, and the national EPI may not have access to this data (A. Colombini, personal communication, August 2016). National EPIs should communicate with institutions overseeing laboratories to obtain information on these expenditures and confirm that surveillance laboratories have received adequate funding. Furthermore, as previously discussed, some countries do not create a budgeted list of surveillance activities when developing their cMYPs, increasing their likelihood of overlooking key resources. When possible, national EPIs should use an ingredients approach to ensure the consideration of all major surveillance inputs.

The quality of surveillance costs reported in cMYPs could also be improved by changes around the use of these tools themselves. To ensure greater accuracy in reported expenditures, surveillance spending estimates in cMYPs should be regularly updated as new information about spending becomes available. While WHO-UNICEF Guidelines do recommend periodic revisions to cMYP data, this needs to be a greater priority for countries [8]. Financing organizations, such as Gavi, could also require countries to report expenditures on all major surveillance cost categories in their cMYPs as a precondition to receiving funds – which could help to ensure that recipient nations have appropriately budgeted for all key surveillance activities.

### 4.1 Limitations

In addition to some potentially missing costs (which may compromise the accuracy of data for certain countries), our analysis encountered several limitations. While WHO provides surveillance recommendations, different countries choose to use different types of surveillance and monitor different numbers of VPDs [16]. National spending on surveillance will differ based on these decisions, which we could not control for in our analysis. We also only considered expenditures from a single baseline year for each country, which may not reflect the country’s “average” level of investment in VPD surveillance (for example, if a country incurred one-time set-up costs during its cMYP baseline year related to the launch of a new form of surveillance). Moreover, our study relied on nations that developed cMYPs – typically low- and middle-income countries. Their governments may consequently receive Gavi and GPEI support for VPD surveillance, and this financing may have influenced the expenditures observed in our analysis.

Several other characteristics of cMYPs also limited our findings. Because cMYPs only account for country-level expenditures, we did not consider regional and global surveillance costs in our calculations. For example, global and regional quality assurance and laboratory certification are key to ensuring VPD surveillance laboratories are performing well, but spending on these activities was not included in our country-level estimates. Another limitation is that cMYP projected costs (for future years) are estimated several years in advance and are often not updated after the tool is published. Although we therefore used baseline (retrospective) data whenever possible, we did use projected costs only to make the Niger case study comparisons. Lastly, cMYPs provide no information on the performance or cost-effectiveness of surveillance systems, as they are designed to help countries with planning and budgeting rather than evaluation [8]. While our analysis indicates which countries spent significantly more than others on surveillance, we cannot conclude that higher-spending countries have stronger surveillance networks or vice versa. Further research is needed to quantify the amount of spending needed to ensure a high-functioning VPD surveillance system in a given context.

## 5 Conclusion

Our findings improve current knowledge of disease surveillance by providing estimates of VPD surveillance expenditures in 63 low- and middle-income countries. In addition to observing large variations in spending, we provide a comparative analysis of cMYP surveillance expenditures between countries and various sources of data. Many countries appear to underestimate surveillance costs due to unclear descriptions in budgets, lack of data, shared health system costs and failure to use an ingredients approach. The miscalculation of surveillance resource requirements may result in underfunded systems for monitoring disease, increasing the risk of not adequately tracking VPDs and not identifying infectious outbreaks among vulnerable populations.

We recommend that international agencies and ministries of health use these results to improve current costing guidelines and practices. To ensure that countries budget for major cost categories, surveillance managers must use clearer descriptions of activities when planning for resource needs. Lastly, more country case studies are needed to assess the cost, quality, and performance of surveillance networks. Through increased knowledge of surveillance costs, countries around the world can better plan for monitoring VPDs and protect populations against communicable illness.

## Footnotes

1 Given the presence of high-spending outlier countries, we chose to report median expenditure values. Mean expenditure values are also available in Table 1 and can be calculated using the data in Appendix 1.
